# Repetition of the Dose

**Published:** 1896-10

**Authors:** B. L. B. Baylies

**Affiliations:** Brooklyn, N. Y.


### REPETITION OF THE DOSE.

(Read before the International Hahnemannian Association, June 1st, 1896.)

B. L. B. Baylies, M. D., Brooklyn, N. Y.

The object of homœopathic prescription is to cure—*certo* "*cito tuto et jucunde.*" And this is possible within the limitations of our broad yet extending domain of symptom-similar drugs. That something more than symptom-similarity is necessary the true votary of Hahnemann's exposition of healing wisdom discovers. He conceives that to prove remedial in many cases the agent must have attained a greater vibratile tension, and strike a more subtile chord of sympathy with the perturbed vital dynamis than can be reached by ordinarily high potencies. He finds when this dynamic symphony is attained the greatest care must be taken in playing upon the human harp, lest the strings be broken or "the sweet bells jangled" and disturbed.

The necessity of repetition and the period of intermission of doses depends upon the activity of the individual medicine and the duration of its action. Jahr states the duration of effect of many medicines administered in the 12th to the 30th potency; of Aconite as eight, sixteen, twenty-four, and forty-eight hours; Belladonna and Bryonia, four to five days; Arsenic-a., thirty-six to forty days; Calcarea and Graphites, fifty days, etc.; the longest action ascribed in general to the minerals.

Second, repetition must be governed by the susceptibility of the patient, which, at present, can only be determined by experiment. The 200ths centesimal have, in my experience in sthenic forms of inflammation or fever, been efficient and well borne in doses repeated every two or three hours. But when the system is poisoned by a chronic dyscrasia manifested in chronic eruptions from the skin or mucous membranes, chronic catarrh, or in malign forms of disease, phthisis, tabes mesenterica, foul ulcers, acute inflammations, complicated with psora in old persons; carbuncles, nervous disorders, malarial fevers, the higher potencies, generally under the hundred thousandth, acted so rapidly and so promptly induced the restorative process that with close observation I have always realized the wisdom of Hahnemann's precept not to repeat the remedy during progress in improvement. In some acute cases of apparent psoric taint I have only been obliged to repeat the forty-five thousandth for a few doses at intervals of three or four hours, and later have given a single dose of the hundred thousandth potency, generally the only one needed. In diphtheria I now give one dose, and if required by interruption of improvement to repeat, give a still higher dose. Aggravation or suspension of reaction does and would, in my opinion, often follow the repetition of the higher potencies; but their curative action is so promptly observed that to the attentive mind there is no temptation to repeat them.

In feeble circulation and in nervous disease with hyperæsthesia of the heart, it seems prudent not to give the highest immediately, but to feel the way through somewhat lower potencies; and in grave forms of disease like diphtheria it appears important to economize the medicinal force, for by so doing we at the same time economize the vital force, and generally not at first to stake a higher potency than the forty-five thousandth, since it is easier in this case to advance than to retreat. There are exceptions to all rules, though according to the late and honored Dr. P. P. Wells a lower potency cannot advantageously follow a higher.

We have to feel our way toward that essential harmony of the medicinal with the physical dynamis, and we need all our finest senses, corporeal and physical, for this tentative progress.

In malarial fever a single high-potential dose will usually efface all the phenomena of the disease; an improvement follows and the paroxysms abate in different cases with varying celerity, like the undulative recession of the sea. The great care necessary not to repeat the dose and retard the cure is in this disease distinctly evident. In disorders caused or complicated by one of the "three miasms" of Hahnemann (*Chronic Diseases*) the action of the single dose and the injury of repetition are manifest. In cases of diffused chronic eczema of several years' duration I found in one patient cured with Causticum, in another with Petroleum, much increased excitation and congestion of the skin when a second dose was given after the lapse of several weeks. The patient recovered, but the cure was retarded by the repetition. In a case of bronchial diphtheria the patient's life was threatened by the repetition of the dose (forty-five thousandth) at three hours' interval for thirty-six hours, though decided improvement had followed the earlier doses, and was saved by one dose of the millionth.

I must ask pardon for the brevity of my paper on this subject. It only seemed necessary to state facts, with some illustration.

